# Hybrid composite polyaniline-nickel hydroxide electrode materials for supercapacitor applications

**DOI:** 10.1016/j.heliyon.2018.e00801

**Published:** 2018-09-25

**Authors:** Janardhan H. Shendkar, Vijaykumar V. Jadhav, Pritamkumar V. Shinde, Rajaram S. Mane, Colm O'Dwyer

**Affiliations:** a S. S. J. E S. Arts, Comm. and Sci. College, Dist. Parbhani, Gangakhed, India; b School of Physical Sciences, Swami Ramanand Teerth Marathwada University, Nanded, India; c Department of Physics, Shivaji Mahavidyalaya, Dist. Latur, Udgir, Maharashtra, India; d School of Chemistry, University College Cork, Cork T12YN60, Ireland

**Keywords:** Materials science

## Abstract

Pristine and nanocomposite (NC) hybrid electrodes of polyaniline (PANI)-nickel hydroxide [Ni(OH)_2_] have been prepared by single and two-step electrodeposition processes, respectively, onto stainless-steel (SS) substrates. Enhanced reversibility and stability of amorphous PANI- Ni(OH)_2_ NC electrodes compared to single electrode materials have been explored. PANI has a nanofibrous morphology, Ni(OH)_2_ has nanoplatelet-type morphology, and the NC electrodes retain an overall nanofibrous morphology. The maximum specific capacitance (SC), obtained from integrated charge under voltammetric conditions, for PANI (electro-deposited for 5 min), NC (electrodeposition of Ni(OH)_2_ for 10 min and 20 min onto PANI electrode surface) and Ni(OH)_2_ (electrodeposited for 10 min) electrodes, are 0.59, 39.06, 32.36, and 113.8 F/g, respectively, suggesting higher electrochemical performance of Ni(OH)_2_ electrode compared to PANI and NC electrodes. The retention in SC values with faster scan rates from 10 to 100 mV/s for PANI, NC (10 min), NC (20 min) and Ni(OH)_2_ are 38.7, 61.1, 52.4, and 29.0 %, respectively, explicitly confirming a higher reversibility in NC electrodes. The retention in SC values with increase of cycle number up to 1000 for PANI, NC (10 min), NC (20 min) and Ni(OH)_2_ electrodes are 34.9, 61.5, 67.5, and 40.7 % respectively, demonstrating higher electrochemical stability of NC electrodes over pure-phase electrodes. Nearly 2.15, 79.36, 66.66 and 406.83 mC/cm^2^ charges on PANI, NC (10 min), NC (20 min) and Ni(OH)_2_ electrodes, respectively, are obtained. Inner to total charge and outer to total charge ratios have been used to explain contributing sites to total charge in pristine and NC electrodes.

## 1 Introduction

Electrochemical supercapacitors (ES), have attracted considerable attention in recent years, as they are capable of providing a higher power density than batteries, in addition to high energy density compared to traditional capacitors, long cyclability and fast charge/discharge capability, which are useful in miniaturized consumer electronic products (where high operating potential is required and energy density is directly proportional to the square of operating potential window) such as memory back-up systems, electrical vehicles and industrial power/energy management etc. [1, 2, 3]. Based on the charge storage mode, they are divided in two categories i.e. electrical double layer capacitors and pseudocapacitors or faradaic capacitors. In an electric double layer capacitor, energy is physically stored by means of ionic accumulation at the electrode/electrolyte interface, whereas in a pseudocapacitor, energy is stored due to reversible surface or near-surface faradaic reactions. As a result, the SC of the ES is proportional to the accessible surface area of the electrode material in contact with the electrolyte ions/redox species. The SC value of the active material can be increased by converting the bulk structure to its nanostructure form which eventually possesses a high surface area and smaller particles (which shorten the path length of ion transfer and release mechanical stress during charge/discharge process) [4, 5, 6, 7, 8]. PANI is a commonly used material for ES applications due to availability of several preparation methods for different morphologies, its low-cost and environmental and chemical stabilities etc. [9]. Due to considerable electronic conductivity and moderate SC value, nanofibers/nanowires of PANI are useful for ES [10]. The structure is based on repeating units of aniline, connected in such a way that forms a backbone of alternating nitrogen atoms and benzene rings. PANI exhibits leucoemeraldine, emeraldine, and pernigraniline states depending upon the degree of oxidation of the nitrogen atoms. Of these, only emeraldine salt is electrically conductive [11]. The main drawback of PANI electrode lies in its lower cycling stability and electrochemical inactivity in alkaline solution [12]. Hence, it is necessary to make composite of PANI with other suitable inorganic nanostructures not only to overcome its limited stability, but also to boost the overall capactiance. NCs of PANI with polymers [13], metals [14], metal oxides [15], carbon [16] and graphite [17] etc., have been studied with an aim to achieve higher SC value and more stability i.e. cyclability.

Among the series of transition metal oxides/hydroxides, nickel hydroxide [Ni(OH)_2_] is one active metal hydroxide used as an ES material. With well-defined redox activity, low-cost, layered morphology and high SC value, it has been extensively studied over the last few decades [18, 19]. However, it suffers weak adhesion between the nickel-based material and the current collector (substrate), leading to the loss of active material and short cycle life as an ES electrode [20]. In order to overcome this drawback, NCs of Ni(OH)_2_ with MnO_2_ [21], Ni_3_S_2_/3D GN (three-dimensional graphene network) [22], graphene oxide [23], CoO/rGO [24], Co_3_O_4_/RGO [25] etc., have been studied with enhanced performance. The pioneering work on composite oxides as electrode materials for supercapacitors, such as Co_3_O_4_@MWCNT nanocable, and CoOOH nanoplates with multi-walled carbon nanotubes have proven high cyclic stability over 10,000 cycles [26, 27].

PANI polymerized from aniline in aqueous acidic solution can be converted to several forms with different electrical properties by acid/base treatments and/or oxidation. When semi-oxidized PANI film is immersed in hydrazine aqueous solution/ascorbic acid (15 h), it generally reduces to leucoemeraldine state and when immersed in ammonium peroxydisulfate solution (15 h), oxidizes into pernigraniline state [28] which can also be protonated by dipping in strong acidic solution and deprotonated by immersing in alkaline solution. The electrochemical oxidation/reduction of PANI electrode can be obtained by cyclic voltammetry (CV) between -0.2 to 0.8 V in the acidic medium. In the anodic electrode potential scan of a CV, PANI demonstrates leucoemeraldine-emeraldine-pernigraniline state sequence, while in the cathoic scan PANI follows the reverse sequence i.e. pernigraniline-emeraldine-leucoemeraldine states. The CV of PANI film in voltage window -0.2 to 0.6 V in alkaline solution is electrochemically inactive but mechanically stable [29]. Thus, the direction of polarization of PANI electrode at specific voltage can be used to convert a particular oxidation state of PANI. As PANI electrode is polarised at negative potential, anionic species are expelled due to electrostatic repulsion, while concentration of cationic species increases [30]. Here, single step and two-step electrodeposition methods were developed to deposit PANI, Ni(OH)_2_ and NC electrodes, respectively. We have investigated the structural, morphological, and electrochemical supercapacitive properties of pristine PANI, Ni(OH)_2_ and PANI-Ni(OH)_2_ NC electrodes.

## 2 Experimental

Aniline, nickel nitrate hexahydrate, sulphuric acid and sodium hydroxide of analytical reagent grade were purchased from Merck and used without further purification. The working electrode i.e. pieces of SS (1 cm × 4 cm) were cleaned with acetone and distilled water ultrasonically, polished with emery paper, and dried in air before each trial. An Epsilion potentiostat was used for electrodeposition under galvanostatic mode in a three-electrode system. The standard three-electrode glass cell consisted of SS as working electrode (on which plastic tape was fixed except on one side 1 × 1 cm^2^ area and contact terminal), a platinum plate of 1.5 cm × 1.5 cm as counter electrode, and a Ag/AgCl reference electrode. The depth and distance of the working electrode in the solution was 1 cm from solution surface as well as from the reference and counter electrode. The aniline monomer solution of 0.5 M and 0.5 M of sulphuric acid were prepared separately in distilled water. In the 20 ml, stirring solution of 0.5 M aniline, 0.5 M sulphuric acid solution was poured drop-by-drop to adjust the _p_^H^ of solution <2. In first step, for PANI thin film electrodeposition on 1 cm^2^ SS, polymerization of the mixed homogeneous 20 ml solution of 0.5 M aniline and 0.5 M sulphuric acid was carried out as mentioned above at constant potential of 0.75 V for 5 min and is represented by (A). The Ni(OH)_2_ thin film electrodes (D), were grown by electrodeposition on a 1 cm^2^ SS surface area in a 20 ml solution of 0.05 M nickel nitrate at a constant potential of -1.0 V for 10 min. Finally, in a second step, PANI-Ni(OH)_2_ NC electrodes were obtained by electrodeposition of Ni(OH)_2_ on previously deposited 1 cm^2^ PANI (electrode) surface for 10 min (is represented by (B)) and 20 min (represented by (C)). All electrodes were immersed in distilled water for 2 min and dried in air at room temperature before further measurements. In summary, electrodes A and D were phase pure PANI and Ni(OH)_2_ whereas B and C were their composites.

The X-ray diffraction (XRD) patterns were obtained by using a (Ultima IV, Rigaku 2500) diffractometer with Cu−Kα radiation in the 2*θ* range of 10–80° with scan rate 0.5 sec/step. The surface appearance and elemental analysis of the electrodes were investigated using field emission scanning electron microscopy (FESEM) and energy dispersive X-ray (EDX) images obtained using a Hitachi S-4800 scanning electron microscope. All the electrochemical properties were studied in 1.0 M NaOH electrolyte solution.

## 3 Results and discussion

The electrodeposition of PANI was carried out at constant potential of 0.75 V for 5 min, since above this potential the pernigraniline (non-conducting) state of PANI is dominant [31]. The electrodeposition of green PANI was obtained from the mixed homogenous solution of aniline and sulphuric acid. At applied potential of 0.75 V in a three-electrode system, a current through the conducting substrate (SS) initiated the deposition of a compact layer/film of PANI i.e. lateral growth. With completion of lateral growth i.e. a compact layer covering the surface of SS by PANI, the aniline monomer continues to grow vertically in 1D on the already present active nuclei sites in compact layer instead of forming new nuclei to form 2D planar thin film-type morphology. The eventual deposited film comprises a nanofiber-type morphology of PANI [32]. The electrodeposition of Ni(OH)_2_ and NC film electrodes from the Ni(NO_3_)_2_ precursor was achieved by reduction of the nitrate ions on the SS/PANI electrode surface to produce hydroxide ions [33]. The local _p_^H^ of solution was increased (on account of the generation of the OH‾ ions) and there was the precipitation of Ni(OH)_2_ at the electrode surface,

(1) NO_3_^−^ + 7H_2_O
+ 8e^−^⇄NH_4_^+^ + 10
OH^−^

(2) Ni^2+^ + 2 OH^−^⇄Ni(OH)_2_

In Ni(OH)_2_ electrodeposition, the concentration of the Ni(NO_3_)_2_ precursor (0.05 M) was low in order to ensure efficient utilization of electrochemically generated OH¯ ions because at high concentration, Ni_4_(OH)_4_^4+^ could be formed at the electrode surface blocking OH¯ ions from the reaction interface before deposition occurs [34, 35]. At the time of electrodeposition of Ni(OH)_2_ onto PANI, PANI undergoes reduction from emeraldine state to leucoemeraldine state with time, as its colour was observed to change from green to greenish yellow or pale yellow.

**Fig. 1:**
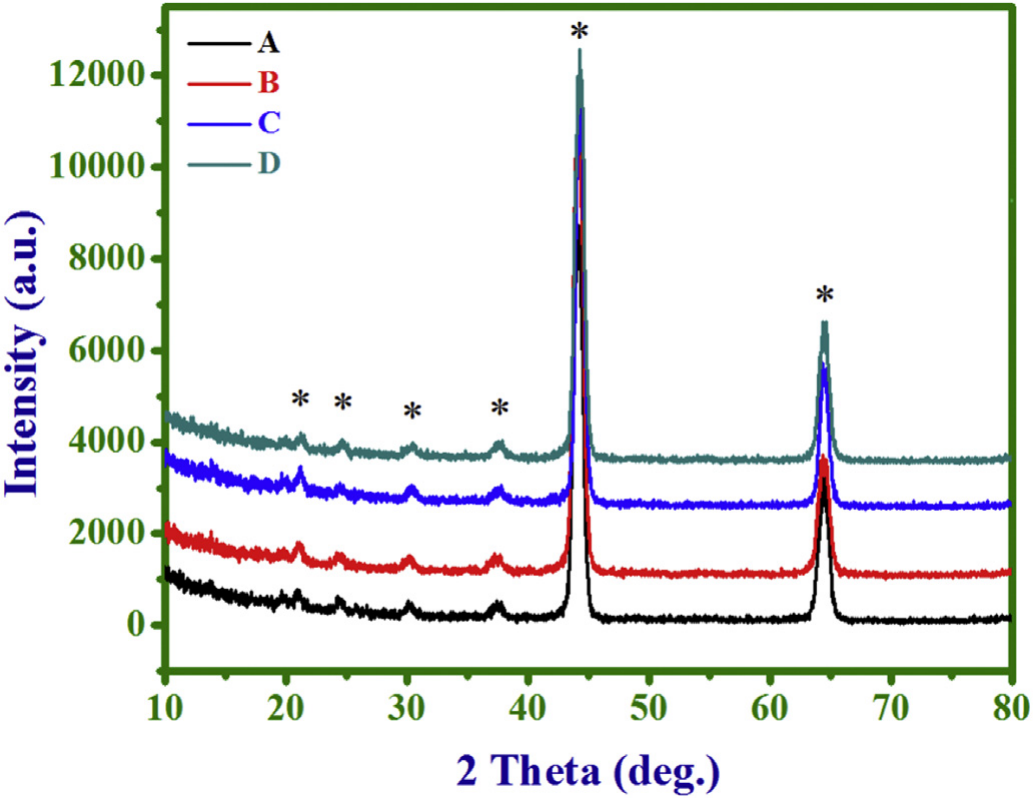
The XRD patterns of PANI (A, 5 min), NC (10 min) (B), NC (20 min) (C), and Ni(OH)_2_ (D, 10 min) electrodes.

Fig. 1 shows the XRD patterns of the PANI and PANI/Ni(OH)_2_ NC and Ni(OH)_2_ electrodes. The peaks with asterisks ‘*’ are of SS. All clearly discernible peaks were from the SS substrate and there was no diffraction from either PANI or Ni(OH)_2_, confirming an amorphous nature.

**Fig. 2:**
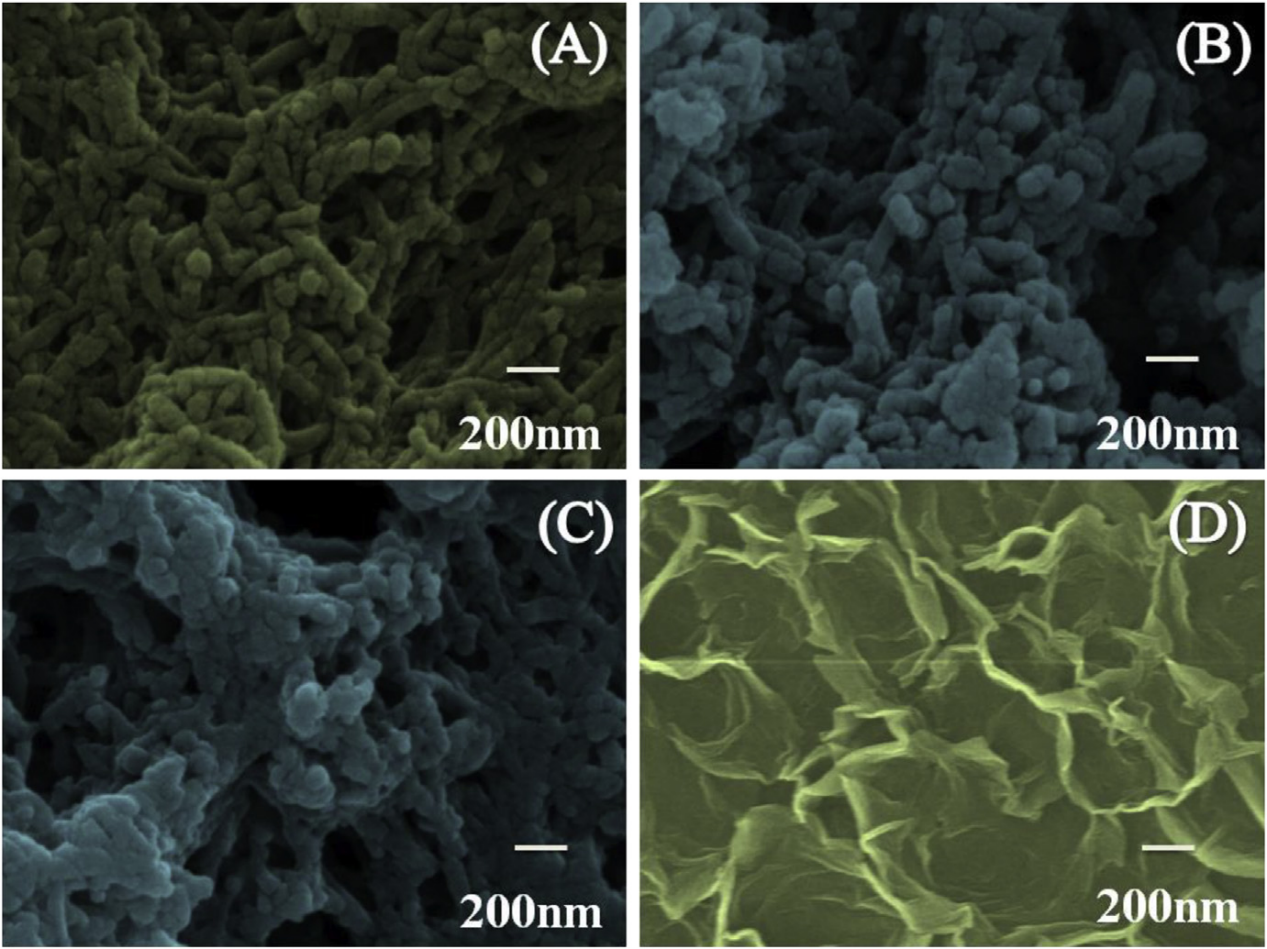
FESEM images of PANI (A), NC (10 min) (B), NC (20 min) (C), and Ni(OH)_2_ (D) electrode surfaces.

**Fig. 3:**
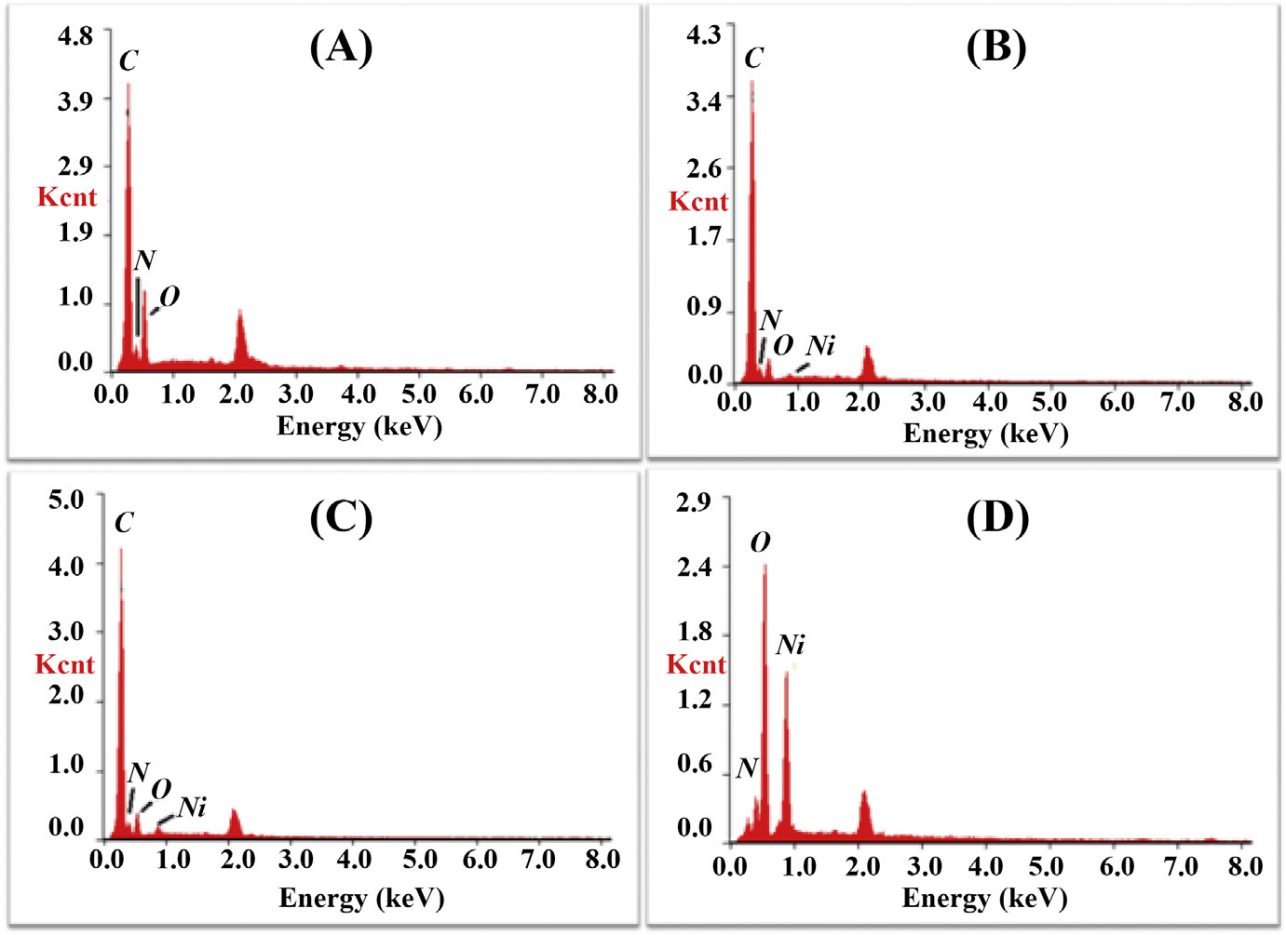
The EDX mapping on PANI (A), NCs (10 min) (B), NCs (20 min) (C) and Ni(OH)_2_ (D) electrode surfaces.

The FESEM images of the PANI, NC for 10 min and 20 min, and Ni(OH)_2_ are presented in Fig. 2 (A–D). The FESEM of PANI [Fig. 2(A)] electrode surface confirms the nanofiber-type morphology. These nanofibers were non-uniform in size and shape. Instantaneous nucleation is a process of nuclei creation at a high rate on a small number of active sites and progressive nucleation is a process of nuclei creation at a low rate but on a large number of active sites. In the present case, the concentration of the aniline was higher (0.5 M), and we assume an instantaneous nucleation process might be dominant. This resulted in increase of current density, which increased the rate of deposition. This process is a likely cause for the increase in length of the nanofibers with time [36, 37]. In Fig. 2(B and C), FESEM images of the NC electrodes of PANI- Ni(OH)_2_ show no considerable change in the fibrous-type morphology, as deposition of Ni(OH)_2_ on PANI was relatively slow due to increased resistance of PANI in neutral medium, as the current was decreased in order to maintain the constant potential drop across the electrode. Fig. 2(D) displays the FESEM image of cathodic electrodeposited Ni(OH)_2_. The prepared electrode exhibited platelet-type surface morphology. Such 2D structure with several wide spaces could be useful for ES application by promoting ionic diffusion/transfer process at the electrode/electrolyte interface. Fig. 3 shows the EDX spectra between energy (keV) and number of Kilocounts (Kcnt) per energy interval of emitted X-rays of PANI (A), (B) NC (10 min), (C) NC (20 min) and Ni(OH)_2_ (D) electrodes. The peaks qualitatively confirm the presence of *C* and *N* of PANI, *C, N, Ni* and *O* of NCs and *Ni* and *O* (and some trace amount of *N* may be on account of non-reduced nitrate ions present in Ni(OH)_2,_ as it was electrodeposited from precursor of Ni(NO_3_)_2_.H_2_O) of Ni(OH)_2_. The peak at ~2.15 keV in all EDX spectra is from the gold coating for imaging.

**Fig. 4:**
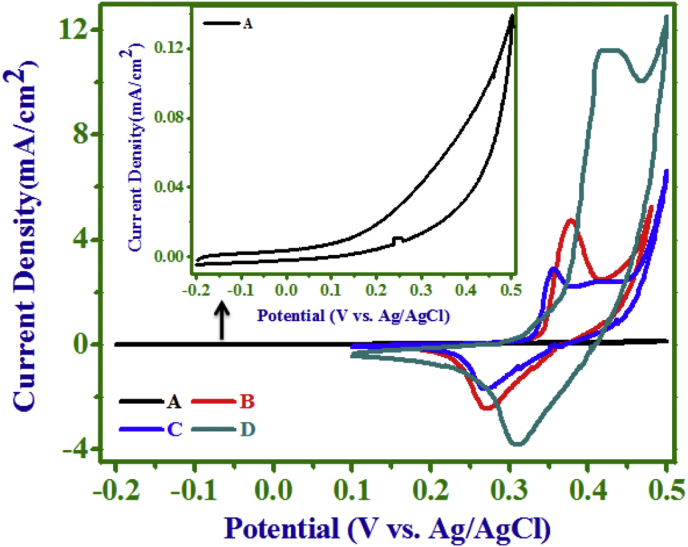
The CV curves of PANI (A), NC (10 min) (B), NC (20 min) (C), and Ni(OH)_2_ (D) electrodes in 1 M NaOH electrolyte (inset is enlarged CV of PANI).

Fig. 4 presents typical CV curves of PANI (A), NCs (B, C) and Ni(OH)_2_ (D) electrodes in 1 M NaOH electrolyte solution at a scan rate of 10 mV/s in the potential range from -0.2–0.5 V for PANI and 0.1–0.5 V for NCs (B, C) and Ni(OH)_2_ (D), while inset in Fig. 4 is an enlarged view of PANI CV. In the CV of PANI an absence of pronounced peak, caused by its inactivity in NaOH electrolyte, contributed extremely low capacitance value. But in the CVs of (B), (C) and (D) electrodes, two strong redox peaks for faradaic redox reactions were confirmed, suggesting the capacitance was mainly due to faradaic redox reactions. Thus, the capacitance of Ni(OH)_2_ electrode in alkaline electrolyte solution was from the charge storage a) in the electric double layer at the electrode/electrolyte interface, and b) in the electrode material by redox reactions on the surface and hydroxyl ion diffusion in the electrode active material [38]. For Ni(OH)_2_, in alkaline medium, the surface faradaic reaction follows [39];

(3) Ni(OH)_2_ + OH ‾⇄NiOOH + H_2_O + e‾

From the observations of CV curves, it was inferred that, this capacitance was mainly from the Ni(OH)_2_ rather than PANI in NC electrodes. The SC values of pristine and NC electrodes were calculated from the areas of CV curves according to equation,

(4) $$C_{s}=\frac{1}{m\nabla V\frac{dV}{dt}}\int I\left(V\right)dV$$

where, *C_s_* is the SC (F/g), A=∫I(V)dV is area of CV (cm^2^), *m* is the mass of active electrode material (g), ∇V is potential window of CV (V) and *dV/dt* is the scan rate (V/s). The SC values calculated at scan rate of 10 mV/s for (A), (B), (C) and (D) electrodes were 0.59, 39.00, 32.36, 113.80 F/g, respectively. The SC value of NC electrode i.e. (B) was higher than NC electrode (C) which was unprecedented. The SC value obtained for Ni(OH)_2_ electrode was greater than that of PANI as well as NC electrodes.

**Fig. 5:**
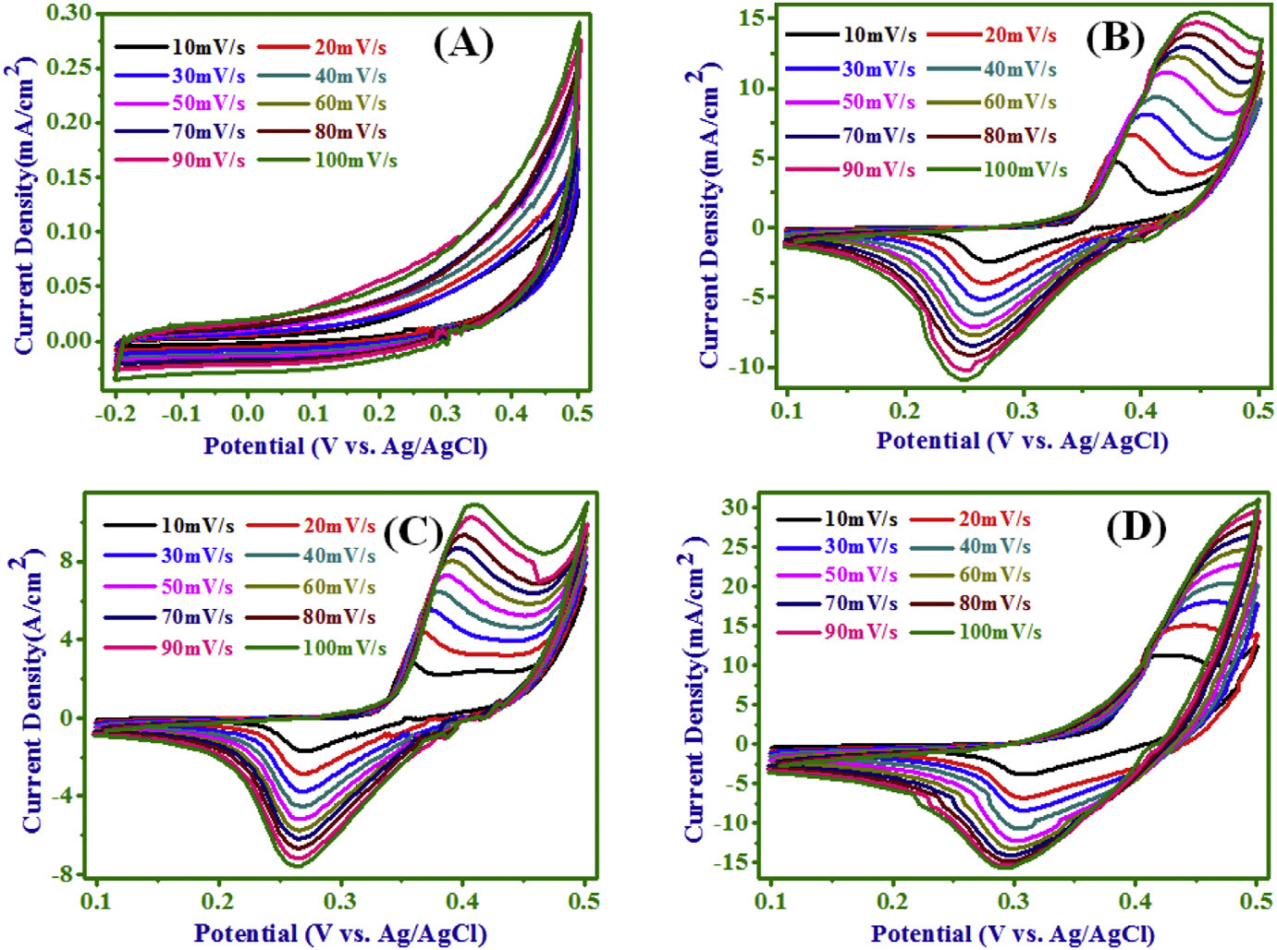
Effect of scan rate on CV of; PANI (A), NC (10 min) (B), NC (20 min) (C), and Ni(OH)_2_ (D) electrodes.

**Fig. 6:**
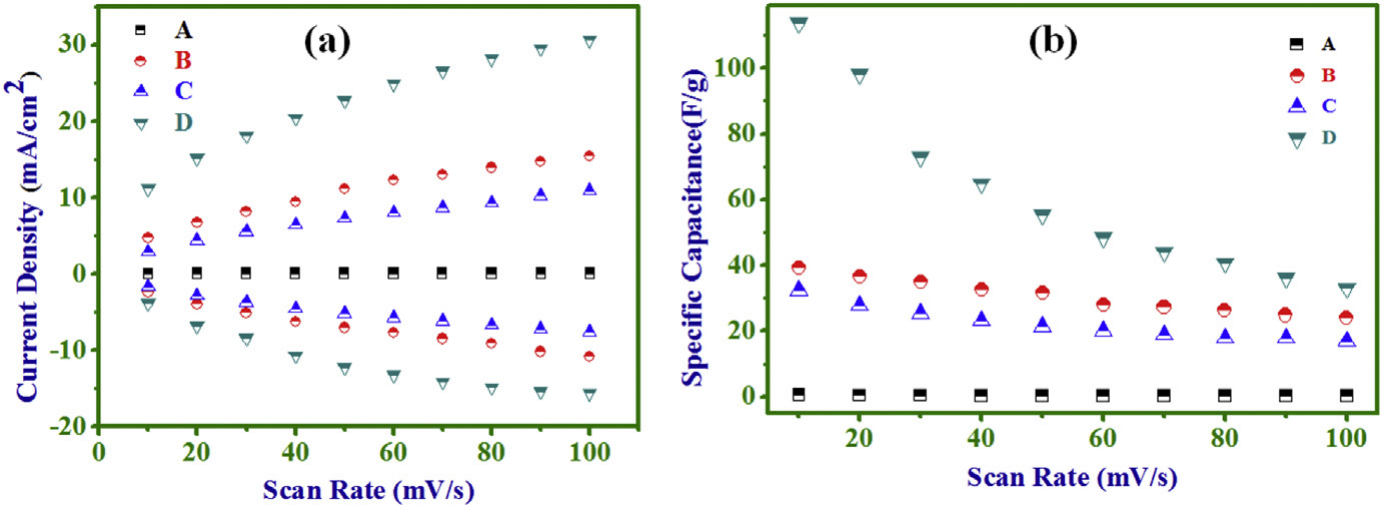
(a) Current density *vs.* scan rate, (b) SC *vs.* scan rate plots of; PANI (A), NC (10 min) (B), NC (20 min) (C), and Ni(OH)_2_ (D) electrodes.

The CVs of pristine and NC electrodes for 10–100 mV/s scan rates are presented in Fig. 5 (A–D). With increasing scan rate, the current density also increased and the oxidation peaks shifted to a more positive potential and the reduction peaks to a more negative potential. This was assigned to an increase of the internal diffusion resistance within the electro-active material with an increase in scan rate [40]. The increase in current density with scan rate was non-linear [Fig. 6(a)], suggesting the accessible active sites of electrode material by electrolyte species (cations/anions) were surface and diffusion-based. The dependence of SC with scan rate is represented in Fig. 6(b), where with increase of scan rate from 10 to 100 mV/s the retention of SC values of A–D electrodes, respectively, were 38.7, 61.1, 52.4 and 29.0%, suggesting higher SC retention in NCs electrodes than both PANI and Ni(OH)_2_ electrodes.

**Fig. 7:**
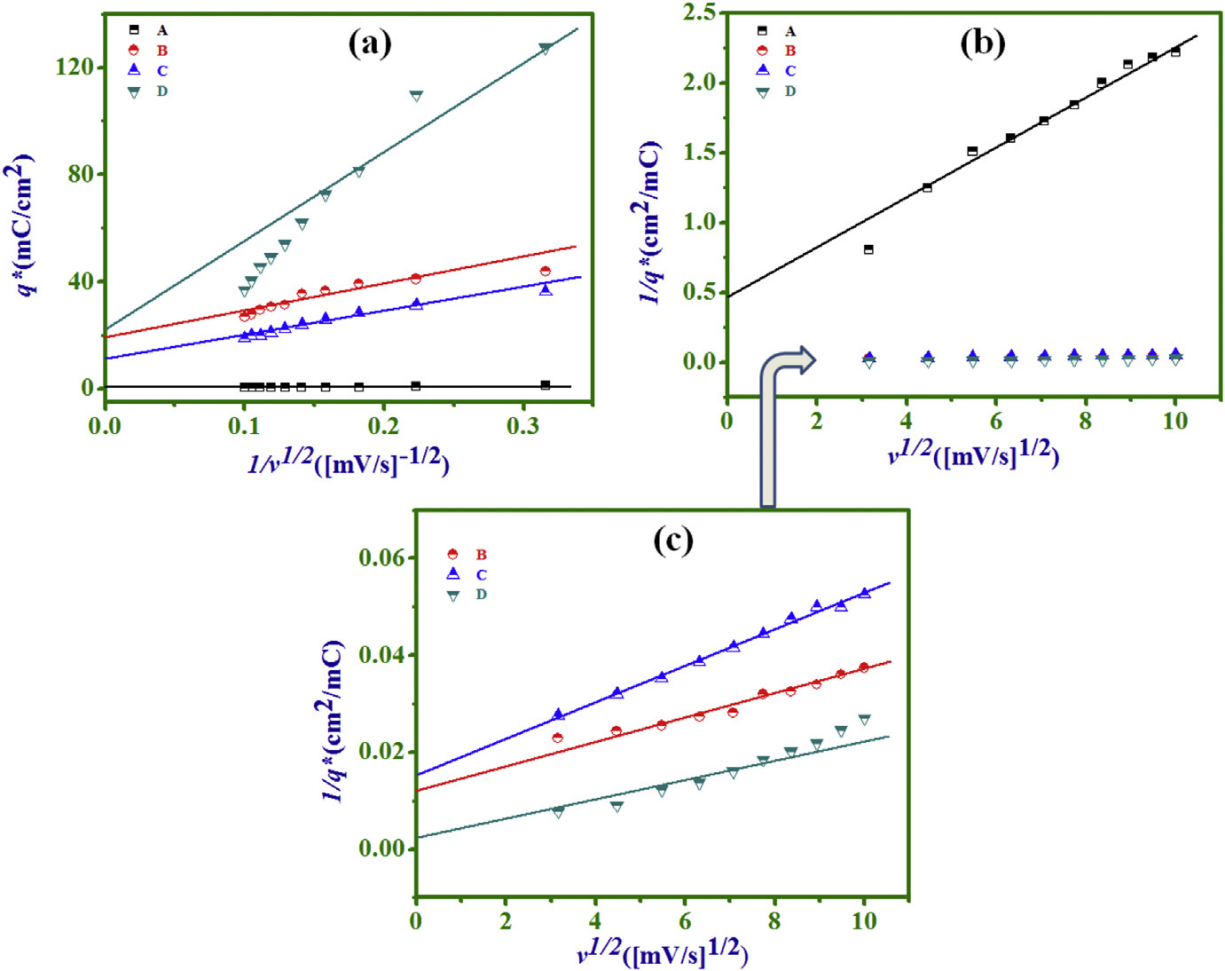
(a) q* *vs.* 1/*v*^1/2^, (b) 1/q* *vs*. *v*^1/2^ plots of; PANI (A), NC-10 min (B), NC-20 min (C), and Ni(OH)_2_ (D) electrodes. (c) Enlarged view of Fig. (b) for (B), (C) and (D) electrodes.

The charge (q*) with a scan rate (*v = dV/dt*) is given by [41];

(5) $$q^{*}=\frac{1}{v}\int I\left(V\right)dV$$

where, ∫I(V)dV represents an area of the respective CV. Fig. 7 illustrates the dependence of charge (q*) on the scan rate to detect the consequence of the cation/anion diffusion resistance on as prepared individual and NCs electrodes. The charge is widely used to quantify the electrochemically active sites of the electrode materials accessed by the aqueous electrolyte that contribute to capacitance [42]. The electrochemically active sites can be categorized as the outer electrochemical active sites that account for outer charge - related to the surface of a thin film, as opposed to the physical outer surface or the entire porous material) (*q*_0_*) contribution and the inner active sites (surfaces of material comprising internal porosity) responsible for inner charge (*q*_i_*) contribution to the total charge. It is known that outer and inner charge contributions come from electrode surface/electrolyte and bulk electrode/electrolyte interfaces, respectively, where for porous materials outer charges contribute less than inner charges, which is opposite in relatively compact materials. The estimation of outer charge (*q*_0_*) can be made by the extrapolation of the charge q* to *v* = ∞ (*v* is a scan rate) from the graph plot of 1/*v* ^1/2^ vs. q* [Fig. 7a]. The estimation of total charge (*q*_t_*) can be obtained by the extrapolation of charge q* to *v* = 0, from the plot of *v*^1/2^ *vs.* 1/q* [43, 44, 45] [Fig. 7b]. The enlarged view of plots for (B), (C), and (D) electrodes are given in Fig. 7 (c), as the variations in Fig. 7(a) are not clearly distinguished. The *q*_i_* is thus defined as the difference between total charge and outer charge. Table 1 represents calculated charges and their charge ratios for four electrodes. The ratios of *q*_i_*/*q*_t_* and *q*_0_*/*q*_t_* represent the contribution of electrochemical charge in the SC due to inner and outer active sites, respectively. From the Table 1 it was concluded that the contribution of total (due to an excess inner charge) charge in D electrode was higher than other electrodes.

**Table 1:** Total, outer and inner charges and their ratios for; (A), (B), (C), and (D) electrodes.

Electrodes	q_t_*mC/cm^2^	q_o_*mC/cm^2^	q_i_*mC/cm^2^	q_o_*/q_t_*	q_i_*/q_t_*
A – PANI	2.15	0.997	1.15	0.464	0.536
B – PANI/Ni(OH)_2_(10 min.)	79.36	19.82	59.54	0.250	0.750
C – PANI/Ni(OH)_2_(20 min.)	66.66	11.39	55.27	0.171	0.829
D – Ni(OH)_2_	406.83	22.66	384.17	0.056	0.944

**Fig. 8:**
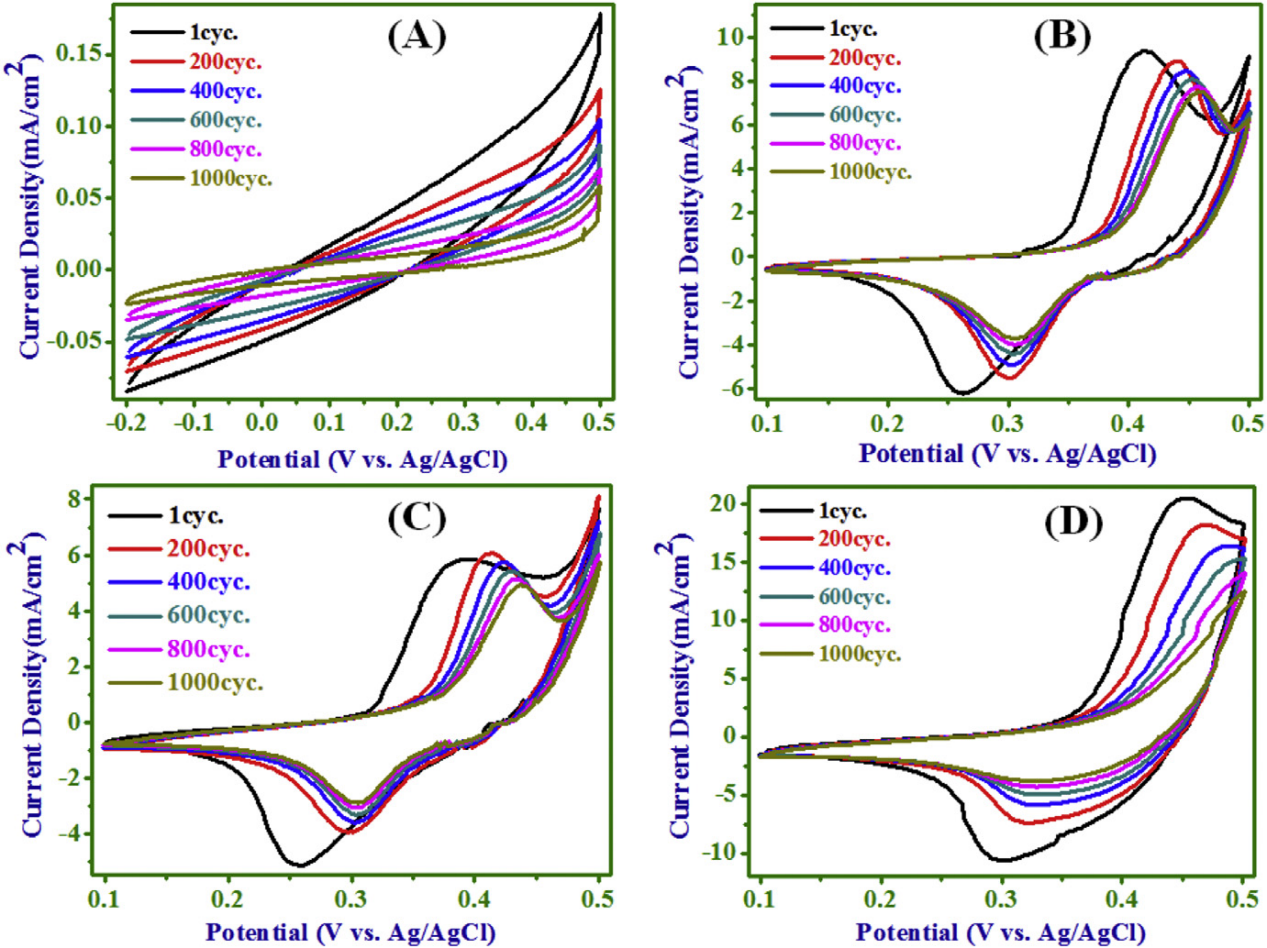
CV of PANI (A), NC-10 min (B), NC-20 min (C), and Ni(OH)_2_ (D) electrodes over 1000 cycles.

**Fig. 9:**
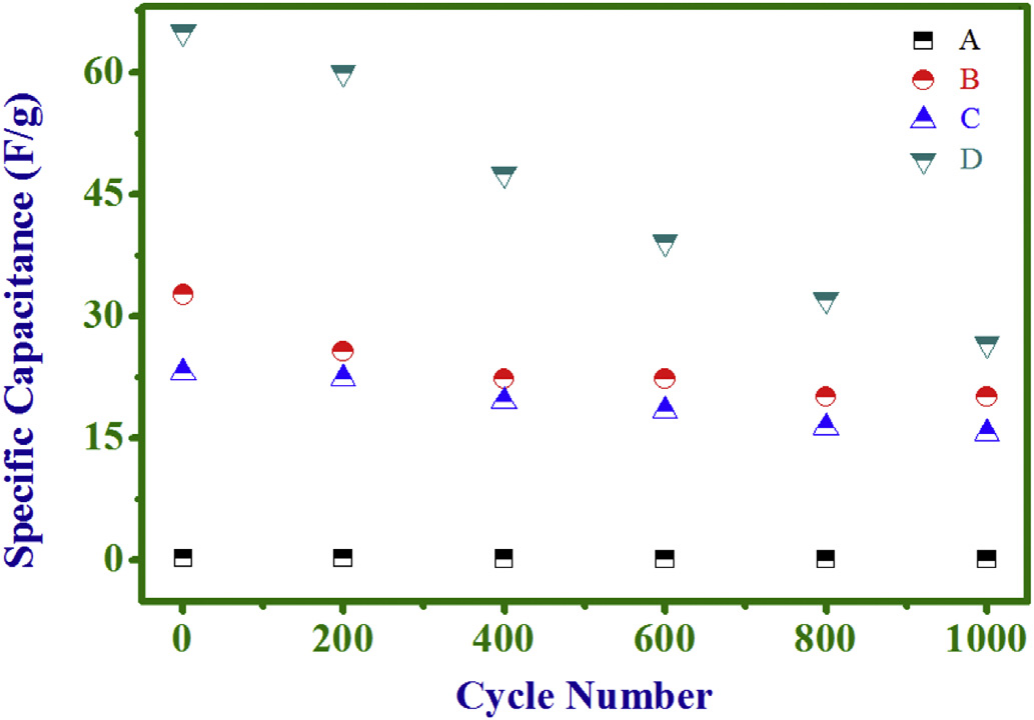
Cycle number dependence of SC of PANI (A), NC (10 min) (B), NC (20 min) (C), and Ni(OH)_2_ (D) electrodes.

As for real supercapacitor operations, besides a high SC value, excellent long term cycle stability has prime importance. The CV (at a scan rate of 40 mV/s) of (A) electrode (-0.2–0.5 V) and of (B), (C) and (D) electrodes (0.1–0.5 V) over 1000 cycles are presented in Fig. 8, confirming chemical stability and mechanical robustness of an individual electrode. The variations of SC as a function of cycle number are given in Fig. 9 for all electrodes. It is seen from data of Fig. 9 that all electrodes could withstand over 1000 cycles with a retention of 34.9, 61.54, 67.5 and 40.7% in SC values of electrodes (A), (B), (C) and (D) respectively, indicating an improvement in the cyclic stability of NC electrodes over the PANI and Ni(OH)_2_ electrodes. The CV curves of NC electrodes (B) and (C) (from Fig. 8) demonstrate well-defined pairs of oxidation/reduction peaks from 1 to 1000 cycles, as compared to Ni(OH)_2_ electrode (D). In the CV of electrodes (B), (C) and (D), the oxidation and reduction peak potentials were both shifted towards more positive potentials. The differences of oxidation and reduction peak potentials of electrodes (B), (C) and (D) for 1 to 1000 cycle were 0.154 to 0.153 V, 0.136 to 0.132 V and 0.152 to 0.17 V, respectively. The potential difference between the oxidation and reduction potentials is a measure of the reversibility of the redox reaction and the relatively smaller values obtained here is an indication of a better reversibility [33, 46, 47] in electrode (B) and (C). In the NC of PANI-Ni(OH)_2_, the fading with cycling is observed due to small but finite difference of oxidation and reduction peak potential of electrodes for 1 to 1000 cycles and expected conducting paths are not provided by PANI networks as it acts as insulator in alkaline solution.

## 4 Conclusions

Room temperature electrochemical deposition synthesis of amorphous PANI, PANI-Ni(OH)_2_ and Ni(OH)_2_ electrodes was carried out. For synthesizing pristine electrodes a single-step electrodeposition method was used and for NC electrodes, a two-step electrodeposition method was applied. Due to electrochemically inactive nature of PANI, its CV area in 1 M NaOH electrolyte and associated capacitance was consierably smaller than those of NC and Ni(OH)_2_ electrodes. The SC values, estimated from the CV areas, of four electrodes PANI (A), NC electrodes (B), (C) and Ni(OH)_2_ (D) measured at 10 mV/s scan rate were 0.593, 39.06, 32.36 and 113.8 F/g respectively. A larger SC in the Ni(OH)_2_ electrode was due to presence of a dominant total charge compared to other electrodes. The NC (B) electrode deposited over a shorter time demonstrated higher SC compared to the NC (C) electrode, which was unexpected.

## Declarations

### Author contribution statement

Janardhan H. Shendkar: Performed the experiments; Wrote the paper.

Vijaykumar V. Jadhav: Conceived and designed the experiments; Analyzed and interpreted the data.

Pritamkumar V. Shinde: Contributed reagents, materials, analysis tools or data.

Rajaram S. Mane: Analyzed and interpreted the data; Contributed reagents, materials, analysis tools or data; Wrote the paper.

Colm O'Dwyer: Analyzed and interpreted the data.

### Funding statement

This research did not receive any specific grant from funding agencies in the public, commercial, or not-for-profit sectors.

### Competing interest statement

The authors declare no conflict of interest.

### Additional information

No additional information is available for this paper.
